# The Role of PI3K Isoforms in Regulating Bone Marrow Microenvironment Signaling Focusing on Acute Myeloid Leukemia and Multiple Myeloma

**DOI:** 10.3390/cancers9040029

**Published:** 2017-03-28

**Authors:** Rachel E. Piddock, Kristian M. Bowles, Stuart A. Rushworth

**Affiliations:** 1 Norwich Medical School, University of East Anglia, Norwich Research Park, Norwich NR4 7UQ, UK; r.piddock@uea.ac.uk (R.E.P.); k.bowles@uea.ac.uk (K.M.B.); 2 Department of Haematology, Norfolk and Norwich University Hospitals National Health Service Trust, Norwich NR4 7UY, UK

**Keywords:** AML, myeloma, microenvironment, PI3K

## Abstract

Despite the development of novel treatments in the past 15 years, many blood cancers still remain ultimately fatal and difficult to treat, particularly acute myeloid leukaemia (AML) and multiple myeloma (MM). While significant progress has been made characterising small-scale genetic mutations and larger-scale chromosomal translocations that contribute to the development of various blood cancers, less is understood about the complex microenvironment of the bone marrow (BM), which is known to be a key player in the pathogenesis of chronic lymphocytic leukaemia (CLL), AML and MM. This niche acts as a sanctuary for the cancerous cells, protecting them from chemotherapeutics and encouraging clonal cell survival. It does this by upregulating a plethora of signalling cascades within the malignant cell, with the phosphatidylinositol-3-kinase (PI3K) pathway taking a critical role. This review will focus on how the PI3K pathway influences disease progression and the individualised role of the PI3K subunits. We will also summarise the current clinical trials for PI3K inhibitors and how these trials impact the treatment of blood cancers.

## 1. Introduction

The phosphatidylinositol-3-kinase (PI3K) pathway has been shown to be constitutively active in the majority of multiple myeloma (MM) and acute myeloid leukaemia (AML) cells [1,2,3] and is critical for the tumour cell growth and survival [4,5,6]. This activation can be attributed to both the cytokines within the bone marrow microenvironment (BMM) and the adhesion of malignant cells to the extracellular matrix [7,8,9]. Moreover, disruption of the PI3K pathway has been shown to cause cell cycle arrest and apoptosis in an assortment of cancers [10,11,12]. Many reviews have highlighted the importance of PI3K in chronic lymphocytic leukaemia (CLL), therefore, this review aims to describe the known individualised roles of the p110 PI3K regulatory subunits (alpha, beta, gamma and delta) in the context of MM and AML. In addition, we will discuss the potential for using PI3K-targeted inhibitors in clinical trials to treat these diseases.

Both MM and AML are haematological malignancies with poor prognoses. Combined, these diseases account for approximately 50,000 deaths per year in the USA [13]. With both AML and MM primarily being diseases of the elderly (average age of diagnosis approx. 67 and 69 years respectively [14]), these malignancies are set to become an ever-increasing problem as life expectancy continues to rise. MM is a cancer of the plasma cell, the terminal differentiation stage of a B-cell, and is characterised by the accumulation of these monoclonal cells within the bone marrow. This can cause the patient to develop osteolytic lesions, immunodeficiency and renal failure [15]. In contrast, AML comprises a group of biologically varied disorders of the haematopoietic myeloid progenitor cells which rapidly results in bone marrow failure.

Despite their physiological differences, both of these malignancies at diagnosis are characterised by the expansion of tumorous cells predominantly within the bone marrow. The presence of tumour cells in the peripheral blood is a poor prognostic factor of both diseases and has been linked to a more aggressive or established malignancy [16,17]. Within the bone marrow, malignant cells have been shown to be protected from chemotherapy and encouraged to proliferate, grow and migrate [18,19,20,21]. Indeed, removal of the cells from this environment into culture results in rapid apoptosis, emphasising the symbiotic relationship between the cancer and the niche in which it proliferates [22,23]. For many patients, current chemotherapies fail to clear the bone marrow of visible disease. Furthermore, even in patients who appear to initially respond well to treatment, a sub-population of cancerous cells defined as minimal residual disease (MRD) may persist and are a primary cause of relapse in this group [24,25,26,27]. In the past 20 years, the development of novel treatments has improved MM/AML patient outcome significantly but despite this progress, resistant or relapsed disease remains inevitable for most. The focus of research is now shifting from the malignancy itself to the beneficial stimuli of the environment in which it resides, with the aim of improving therapies with reduced toxicities and ultimately reducing MRD and increasing time between relapse.

## 2. Phosphatidylinositol 3-Kinase (PI3K) Activation in Cancer

**Figure 1:**
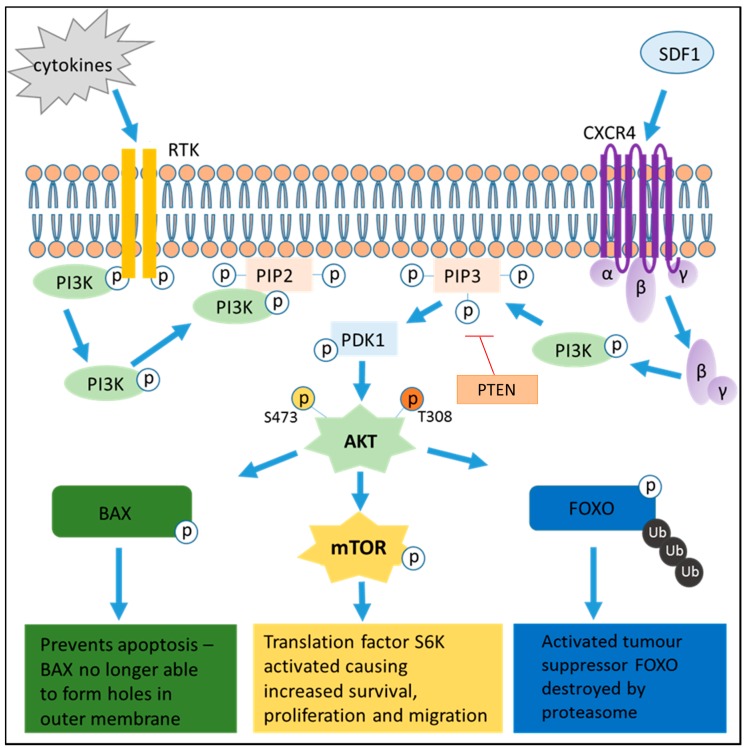
Schematic representation of the phosphatidylinositol-3-kinase (PI3K) pathway. PI3K is phosphorylated via receptor tyrosine kinases (RTKs) and G-protein coupled receptors (GCPRs) located in the cell surface membrane. This causes activation of AKT and its downstream targets, resulting in increased cell survival and migration.

PI3Ks are known to aid the regulation of many differing cell functions, including survival and proliferation. The incongruous activation of the PI3K pathway is common to many cancers, and is well described in MM and AML. When activated, PI3K is able to phosphorylate PIP_2_, a phospholipid component of the cell membrane, to become PIP_3_ (Figure 1). PIP_3_ acts as a docking site for proteins with pleckstrin-homology (PH) domains, which includes the master kinase Phosphoinositide-dependent kinase 1 (PDK1) and its downstream target AKT (also known as protein kinase B). AKT can then activate a plethora of pro-survival signalling cascades, resulting in a reduction in apoptosis and increase in cell motility, survival and growth. Under typical conditions, the lipid phosphatase PTEN (phosphatase and tensin homolog) acts as a negative regulator of the PI3K pathway, de-phosphorylating PIP_3_ and preventing AKT activation-effectively “turning off” the PI3K pathway. Loss of PTEN functionality has been reported in several cancer types, further enhancing the pro-tumoural effect of the PI3K pathway and correlating with a more aggressive disease phenotype.

There are three classes of PI3Ks, with class I being most frequently linked with cancer development. This class of PI3Ks consists of a regulatory subunit and one of four catalytic subunits: p110α, p110β, and p110δ (Class 1A) and p110γ (Class 1B). Whilst a relatively low percentage of PI3K or PTEN mutations are observed in both MM and AML cells [28,29], all four of these isoforms have been shown to be overexpressed in cell lines and primary tissue. p110α and p110β subunits are expressed ubiquitously in cells. Furthermore, p110δ and p110γ are known to be specifically enriched by the haematopoietic system—primarily in leukocytes [30]. This is of interest when considering haematological malignancies as it potentially provides a more tissue-specific, localised target for the treatment of white blood cell malignancies with fewer side effects for patients. Below, we have separated the individual PI3K subunits into sections in order to discuss suitability as a target in blood cancers.

### 2.1. p110α

p110α has long been associated with many different cancers, including cervical [31] and breast cancer [32]. Increased copy number of the PIK3CA gene and subsequent 3q26.3 amplification was found in a significant percentage of primary tissues in these studies, however this is the only PI3K subunit for which a somatic activation has so far been identified [33,34]. This mutation can often be found in concordance with a loss of PTEN functionality—most commonly in breast, endometrial and colon cancers [35]—further increasing the role of the PI3K pathway in tumorigenesis. Despite this mutation being so frequent in solid tumours, it is uncommon in plasma cell disorders and haematological malignancies in general and is only seen at the most advanced stages of plasma cell leukaemia [36]. Recently, it has been shown that inhibition of p110α on its own may not be effective in Luminal Breast Cancer, due to a compensatory rapid accumulation of PIP_3_ produced by the p110β isoform [37]. This activation, however, did not consistently restore AKT phosphorylation, raising questions about the reliability of phospho-AKT monitored PI3K activation.

### 2.2. p110β

p110β has been linked to platelet aggregation [38] and is unique in that it has been shown to be activated by both receptor tyrosine kinases (RTK) and G-protein coupled receptors (GCPR) [39]. Mutations in the PIK3CB gene are rare, however increased expression of this gene has been observed in prostate cancer [40], with knockdown (KD) of this gene resulting in increased apoptosis and cell cycle arrest in stage G1. p110β has been shown to be required to maintain PI3K signalling in PTEN-deficient cancer cells, indicating the opportunity for isoform-specific inhibition in the treatment of this cancer type [41]. In MM, the necessity for p110β is not yet established—with knockdown (KD) of this gene so far reported as proving to be insignificant in terms of cell survival [42]. AML cells however have been shown to become sensitised to chemotherapeutics upon p110β KD, with significant reduction in cell viability [43].

### 2.3. p110δ

Alongside p110γ, p110δ plays a critical role in haematological malignancy pathogenesis and in addition has been linked to various immune disorders, such as PI3Kδ syndrome and inflammatory bowel disease [44,45]. p110δ has a critical role in B-Cell development and has been shown to have an oncogenic role in a number of blood cancers, including CLL, acute lymphoblastic leukaemia (ALL), MM and AML [6,46,47]. This has led to the development of p110δ-specific inhibitors, discussed below, that have been shown to slow and reduce tumour growth, even in the presence of protective bone marrow microenvironment (BMM) signalling.

### 2.4. p110γ

p110γ has been shown to have a critical role in GCPR-mediated PIP_3_ production, which in turn strictly controls cell motility in macrophages [48]. Mutation of the PI3KCG gene has previously been implicated in lung cancer [49] and inhibition of the p110γ subunit resulted in reduced proliferation of these cells in idiopathic pulmonary fibrosis [50]. Considering this isoform’s prominent role in macrophage motility, this may provide a therapeutic target in AML.

## 3. PI3K Pathway Activation

The PI3K pathway has been shown to be activated via a wide assortment of receptor tyrosine kinases (RTKs)—including platelet-derived growth factor receptor (PDGFR) [51,52], c-MET [53], insulin-like growth factor-I receptor (IGF-IR) [54] and Fms-Related Tyrosine Kinase 3 (FLT3) [55]. Constitutive activation of these RTKs (either by mutation/duplication of the RTK such as that reported in AML [56] or overexpression of its complimentary ligand) results in the upregulation of this pathway and has been associated with poor patient prognosis in many cancer types [57,58]. As previously mentioned, G-coupled protein receptors have also been linked to PI3K activation, however this is specific to the p110δ/γ subunits and is not yet well defined. Micro-environmental factors that have been shown to directly contribute to both MM and AML pathogenesis (Table 1) have also been associated with PI3K pathway upregulation, inferring the presence of a positive feedback loop. Of these, Interleukin-6 (IL-6) and Stromal-Derived Factor-1 (SDF-1) are arguably the most significant in MM and AML pathogenesis and have been shown to be stimulated by tumour cell/BMM cell interactions. This special relationship is discussed in more detail below.

## 4. The Protective Effect of the Bone Marrow Microenvironment

The bone marrow microenvironment comprises a plethora of cell types, including macrophages, fibroblastic stromal cells and endothelial cells [59]. In addition to these cells, there is also a vast set of growth factors, cytokines and hormones that contribute to the complex haematopoietic process. The physiologic balance of these soluble factors is influenced by the presence of malignant cells in both AML and MM which results in biological changes within the stromal cells (as well as the malignant cells themselves) to benefit disease progression [60]. This pro-tumoural environment promotes activation of signalling cascades within both benign and malignant cells which inhibit apoptosis, and promote processes such as proliferation and angiogenesis. Many of these soluble factors upregulate the PI3K pathway, a summary of which is shown in Table 1.

IL-6, for example, is critical in MM disease progression, with IL-6 deficient mice unable to develop MM [61] and elevated IL-6 serum levels being associated with poor patient prognosis [62]. In AML, in addition to PI3K pathway activation, IL-6 has been shown to activate the Jak/Stat pathway via Stat3 [63]—a pathway that is known to be commonly dis-regulated in a sub-set of AML patients [64]. Therapies that abrogate IL-6 triggered cascades (such as monoclonal antibody therapy [65]) have been shown to help overcome chemotherapy resistant sub-populations of malignant cells. These agents have also achieved remarkable efficacy in clinical trials in both relapsed/refractory MM [66] and AML [67]. Malignant cells are also known to be protected in the BMM when adhered to the extracellular matrix [68,69,70,71] (collagen, fibronectin and laminin) or to bone marrow mesenchymal stromal cells (BM-MSCs) [72]. Interestingly, our group has shown that activation of BM-MSC by AML co-culture is not inhibited by the pan-PI3K inhibitor LY294002 [73], thus suggesting that PI3K inhibition in the context of the malignant BMM will not have adverse effects on the surrounding environment. However, these results are from in vitro co-cultures and the translation to in vivo models needs to be further investigated.

Stromal-derived factor 1 (SDF-1), also known as CXCL12, is present in the BMM in high concentrations. It is produced by stromal cells and is a chemo-attractant to both AML and MM cells, causing them to ‘home’ to the bone marrow [74,75]. As well as mediating cell migration, SDF-1 has been shown to promote haematopoiesis, increasing the proliferation rates of CD34+ cells [76]. Upon binding, MM cells have been shown to undergo growth arrest [77] as well as the upregulation of pro-survival pathways and secretion of cytokines that are beneficial to both the cancerous cells and the environment on which they depend. AML cells too are known to depend on this adhesion, becoming sensitised to chemotherapeutics upon antagonising CXCR4 [78].

In vitro 3D tissue culture techniques provide a potential improvement to traditional 2D cultures as they can provide a scaffold on which to grow extracellular components and can be utilised to study the effect of malignant cells in an environment similar to the BM [79]. These technologies offer an alternative to 2D cultures but do not mimic the biological system of the murine models that are currently used to understand the microenvironment in the pathogenesis of MM and AML. The complex nature of the BMM and its interactions with haematological malignancies is an area under intense investigation as we and others believe that targeting the environment could well be a means of eradicating the tumour. There are currently two types of animal models routinely used in the study of blood cancers: (1) injection of malignant cells into immune deficient mice (such as NOD.Cg-Prkdcscid Il2rgtm1Wjl/SzJ (NSG) mice); and (2) the injection of allogenic malignant cells into C57BL/6 mice. A third model has recently been proposed in which a scaffold is implanted into immune deficient mice that comprises the patient’s microenvironment and the malignant cell [80]. There are, however, several limitations of these models when considering the BMM. In the immune deficient mice, the absence of a functional immune system and its interactions with the human malignancy is not representative of a natural tumour environment. Subsequently, compounds that may have shown promise in a pre-clinical setting do not translate well into clinical trials [81].

When looking at allogenic tumour models, it is pertinent to understand that species–species differences do occur. For example, telomerase (which is for the most part inactive in adult human cells) is still active in a murine model, suggesting that murine cells are more likely to undergo malignant transformation [82]. In addition, the gene that codes for the cytokine IL-8 (which is critical for MM disease progression and osteoclastic differentiation [83]) is not present in the murine genome [84]). New models are always in development, and recent work has been done to ‘humanise’ the mouse bone marrow, with the aim of replicating the natural microenvironment [85]. Briefly, BM-MSCs can be used to reform the bone marrow of a radiation depleted NSG mouse on which a patient’s malignant cells are transplanted resulting in ‘XactMice’ or ‘xenochimeric’ mice. This step of essentially providing the mice with a bone marrow transplant should make the model a more accurate reflection of a human immune system.

## 5. Clinical Implications

Scientific observations on PI3K signalling in cancer have resulted in the development of a number of PI3K pathway inhibitors over the past 15 years, including isoform-specific inhibitors, which are now in clinical trials across a spectrum of haematological malignancies (a selection of recent trials is highlighted in Table 2). The first wave of PI3K pathway inhibitors to reach the clinic were rapalogues (rapamycin and its analogues) which work via inhibiting mTOR, a downstream target of PI3K activation. Although these drugs were effective in several forms of neoplasms (including neuroendocrine tumours [112] and mantle-cell lymphoma [113]), their use as a single agent treatment was limited due to compensatory pathway activation and subsequent resistance. Rapalogue development was closely followed by the use of small molecules that bound to the catalytic site of mTOR [114] and dual pan-class I PI3K/mTOR inhibitors. These second-generation compounds provided an advantage as they could target the PI3K pathway at two points, suppressing both mTORC1/C2 and PI3K itself [115].

The PI3K/Akt pathway has previously been implicated in the stabilisation of Hypoxia inducible factor 1α (HIF-1α) which is a key regulator in metabolic adaption to low oxygen [116]. This is achieved via the activation of heat shock proteins (Hsp) including Hsp90 and Hsp70, which are known to act in conjunction as molecular chaperones, and have been shown to be over-expressed in many human cancers [117,118,119]. The acute role of Hsps has been described in both AML and MM, where they have been shown to be critical for malignant cell growth and survival [120,121]. Interestingly, AML blasts with a FMS-like tyrosine kinase 3 (FLT-3) mutation are highly sensitive to the Hsp90 inhibitor Ganetespib [122,123]. Inhibiting the PI3K pathway therefore has the potential to reduce the activity of Hsps, especially in the sub-population of patients with FLT3 mutations.

Pan-PI3K inhibition, although effective, can have dose-limiting and adverse effects including hyperglycaemia and varying gastrointestinal conditions. When used in the treatment of breast cancer, clinical studies showed that approximately 60% of patients experienced stomatitis with immunosuppression and non-infectious pneumonitis also presenting at high frequency (40% and 15% respectively) [124]. The inhibition of the specific PI3K subunits therefore provides a far more attractive and directed option, especially when considering elderly patients. Isoform-specific inhibition would be more easily tolerated in combination with traditional chemotherapeutics and could be tailored to the individual nature of the disease. As previously mentioned, p110α and p110β are expressed ubiquitously in many different tissue types, however, p110δ and p110γ are mainly expressed in haematopoietic cells. Targeting just the p110δ and p110γ isoforms could therefore be beneficial for the treatment of haematological malignancies. Consequently, several isoform-specific PI3K inhibitors have now completed phase III trials for haematological malignancies and have started appearing in clinics worldwide. Idelalisib (p110δ inhibitor), for example, is currently being used in combination with rituximab for the treatment of follicular lymphoma [125], and was previously used for treating CLL [126]. In respect to MM and AML, it would be of great benefit to use these isoform-specific PI3K inhibitors to aid in severing the protective effects these malignant cells experience in the bone marrow.

The full potential of PI3K inhibitors as a complementary therapy has not yet been realised. One interesting combination is the use of PI3K inhibitors in conjunction with programmed cell death protein 1 (PD-1) inhibitors. PD-1 is expressed on activated T-lymphocytes and B cells [127], and inhibition of this receptor effectively results in the immune system ‘attacking’ malignant cells [128]. However, PD-1 inhibition has been shown to decrease the activity of the PI3K suppressor PTEN, subsequently causing an increase in PI3K pathway activity [129]. PI3K inhibitors could therefore provide relief for this off-target effect and increase the efficacy of PD-1 inhibitors. Despite the plethora of clinical trials involving PD-1 inhibition, there is currently only one phase I clinical trial looking at the combination of PD-1 inhibitor and PI3K inhibitor and this is in solid tumours (NCT02646748). It would be of great interest to see how this inhibition translates into haematological malignancies.

There are, however, drawbacks when considering the PI3K pathway as a therapeutic target. For example, the PI3K pathway has significant overlap with parallel cascades and pharmacological inhibition may result in the activation of these compensatory pathways. These include processes such as NOTCH-MYC amplification [130] or ERK pathway activation [131], reducing the efficacy of treatment. The biomarkers that predict both susceptibility and resistance to PI3K pathway inhibition will also need to be identified, which when considering cancers with multiple sub-populations is highly complex. Also, the current range of PI3K inhibitors lack sensitivity to mutant isoforms and the therapeutic opportunities may therefore be limited. In respect to MM and AML, it would be of great benefit to use these isoform-specific PI3K inhibitors to aid in disrupting the protective effects that these malignant cells experience in the bone marrow. This should increase the efficacy of current chemotherapies and increase the duration of patient remission.

## 6. Conclusions

The PI3K pathway is a major player in terms of cancer development, however the potential individualised roles of its catalytic subunits are yet to be fully defined, with only a small fraction of clinical trials focussing on the PI3K isoforms. The central role of PI3K signalling in many cell types, not just in tumorous tissue, highlights the need for specific subunit inhibition, with the expectation that such a strategy would limit off-target effects and increase the tolerated dosages in vivo. The majority of key cytokines involved in AML and MM pathogenesis have been shown to activate the PI3K pathway within the bone marrow microenvironment, accounting for the aberrant activation observed in patient samples where no somatic mutations are present. As PI3K isoform-specific inhibitors enter clinical trials, it remains to be seen the optimal co-therapies with which these drugs are best administered. Finally, as p110δ/γ are primarily expressed by leukocytes and the inhibitors of these subunits (such as those recently investigated in CLL and lymphoma [132]) have the potential to reverse some of the protective effects of the BMM, it appears that isoform-specific inhibition of p110δ and/or γ hold the most promise in haemic malignancies.

## Figures and Tables

**Table 1:** Common cytokines and soluble factors involved in the pathogenesis of haemic malignancies that are also implicated in PI3K/Akt pathway activation.

Soluble Factor	Involvement in MM Pathogenesis	Involvement in AML Pathogenesis	Involvement in PI3K/Akt Pathway
APRIL	Survival factor for BM plasmablasts [86]		[87]
BAFF	Normal plasma cell development [88]		[87,89]
IGF-1	Induces growth in all MM cell lines, promotes cell cycle progression [90,91]		[92]
IL-6	Promotes drug resistance, high levels associated with poor prognosis [93,94]	Upregulates STAT3 increasing AML proliferation and survival [63]	[95,96]
IL-8	Increases osteoclastogenesis and promotes angiogenesis [97]	Increases survival, invasion and proliferation of AML cells [98]	[99]
MIP-1a	Increased survival and osteoclast production [100]		[101]
SDF-1	Mediates MM homing to the BM; tumour growth; drug resistance [74]	Mediates AML migration, BTK shown to be involved [102,103]	[104]
TNFα	Induces expression of pro-survival factors [105]	Stimulates AML blast growth via colony stimulating factor [106]	[107]
VEGF	Angiogenesis, promotes MM survival and attenuates apoptosis [108]	Upregulated, increases rate of angiogenesis [109,110]	[111]

**Table 2:** Recent PI3K pathway inhibitors in clinical trials for the treatment of common blood cancers. Data taken from ClinicalTrials.gov.

Phase	Status	Drug Name	Target	Mono/Co Therapy	ClinicalTrials.Gov. Number
I	Completed	Idelalisib	p110δ	Mono	NCT01555281
I	Recruiting	CUDC-907	p110α + HDAC1/2/3/10	Mono	NCT01742988
I/II	Completed	Afuresertib	Akt	Co	NCT01476137
I/II	Recruiting	Nelfinavir	pan PI3K	Co	NCT01555281
I/II	Recruiting	ACP-319	p110δ	Co	NCT02328014
Ib/II	Completed	BYL719	p110α	Co	NCT02144038
II	Recruiting	Gedatolisib	p110α/γ + mTOR	Mono	NCT02438761
III	Ongoing, not recruiting	Duvelisib	p110δ/γ	Co	NCT02004522
